# PEPTIDOGLYCAN RECOGNITION PROTEIN-1 PREDICTS CHEMOTHERAPY-INDUCED CARDIOVASCULAR DYSFUNCTION

**DOI:** 10.1093/geroni/igad104.0092

**Published:** 2023-12-21

**Authors:** Valentina Todorova, Gohar Azhar, Jeanne Wei

**Affiliations:** University of Arkansas for Medical Sciences, Little Rock, Arkansas, United States; University of Arkansas for Medical Sciences, Little Rock, Arkansas, United States; University of Arkansas for Medical Sciences, Little Rock, Arkansas, United States

## Abstract

Doxorubicin (DOX) is an effective anti-cancer agent but may induce cardiotoxicity and cognitive dysfunction through inflammatory mechanisms. Peptidoglycan recognition protein 1 (PGLYRP1), secreted by inflammatory cells such as neutrophils has been elevated in the circulation of patients with cardiovascular diseases such as myocardial infarction and atherosclerosis. We hypothesized that PGLYRP1 might be elevated in cancer patients receiving DOX and will correlate with cardiac or cognitive function. Gene expression of peripheral blood cells and plasma protein concentration of PGLYRP1 were determined by reverse transcription-quantitative polymerase chain reaction (RT-QPCR) and ELISA in 18 breast cancer patients with subclinical cardiotoxicity and in some of the cases with self-reported memory problems in comparison with 22 patients without cardiotoxicity. After adjusting for age and race using multivariate logistic regression, we found a positive correlation between PGLYRP1 log2 fold change of gene expression (0.878 versus 0.534, p=0.05) and protein expression (0.920 versus 0.534, p=0.08) in patients with DOX-induced cardiotoxicity, as evident by left ventricle ejection fraction (LVEF) reduction by >10% versus baseline. Reduced LVEF secondary to DOX, appeared to cause memory impairment similar to vascular cognitive decline. The results from this study suggest that PGLYRP1 could potentially be used as predictive biomarker for DOX-induced cardiotoxicity and Alzheimer’s disease related dementias (ADRD). The role of PGLYRP1 in DOX -associated cardiac and cognitive decline in older cancer patients needs further exploration in larger studies.

